# VPS13D affects epileptic seizures by regulating mitochondrial fission and autophagy in epileptic rats

**DOI:** 10.1016/j.gendis.2024.101266

**Published:** 2024-03-19

**Authors:** Jian Wang, Fan Zhang, Zhong Luo, Haiqing Zhang, Changyin Yu, Zucai Xu

**Affiliations:** a Department of Neurology, Affiliated Hospital of Zunyi Medical University, Zunyi, Guizhou 563000, China; b Department of Neurology, Affiliated Aerospace Hospital of Zunyi Medical University, Zunyi, Guizhou 563000, China; c Department of Clinical Medicine, Zunyi Medical and Pharmaceutical College, Zunyi, Guizhou 563000, China

**Keywords:** Autophagy, Mitochondrial dynamics, Mitochondrial fission, Seizures, VPS13D

## Abstract

Abnormal mitochondrial dynamics can lead to seizures, and improved mitochondrial dynamics can alleviate seizures. Vacuolar protein sorting 13D (VPS13D) is closely associated with regulating mitochondrial homeostasis and autophagy. However, further investigation is required to determine whether VPS13D affects seizures by influencing mitochondrial dynamics and autophagy. We aimed to investigate the influence of VPS13D on behavior in a rat model of acute epileptic seizures. Hence, we established an acute epileptic seizure rat model and employed the CRISPR/CAS9 technology to construct a lentivirus to silence the *Vps13d* gene. Furthermore, we used the HT22 mouse hippocampal neuron cell line to establish a stable strain with suppressed expression of *Vps13d in vitro*. Then, we performed quantitative proteomic and bioinformatics analyses to confirm the mechanism by which VPS13D influences mitochondrial dynamics and autophagy, both *in vitro* and *in vivo* using the experimental acute epileptic seizure model. We found that knockdown of *Vps13d* resulted in reduced seizure latency and increased seizure frequency in the experimental rats. Immunofluorescence staining and western blot analysis revealed a significant increase in mitochondrial dynamin-related protein 1 expression following *Vps13d* knockdown. Moreover, we observed a significant reduction in LC3II protein expression levels and the LC3II/LC3I ratio (indicators for autophagy) accompanied by a significant increase in P62 expression (an autophagy adaptor protein). The proteomic analysis confirmed the up-regulation of P62 protein expression. Therefore, we propose that VPS13D plays a role in modulating seizures by influencing mitochondrial dynamics and autophagy.

## Introduction

Epilepsy is a chronic brain dysfunction syndrome characterized by highly synchronized abnormal brain neuronal discharges. Epilepsy is a significant global public health challenge affecting diverse ethnic groups worldwide.^1^ The global prevalence of epilepsy is estimated to be 0.5%–1%, with approximately 70 million individuals affected, and among them, 75% experience active epilepsy.^2^^,^^3^ In China, according to incomplete statistics, there are approximately 10 million patients with epilepsy, and this number continues to rise annually by an average of approximately 400,000–600,000 cases.^4^^,^^5^ Standardized treatment effectively controls seizures in approximately 70%–80% of patients; however, approximately 20%–30% experience intractable epilepsy.^6^ Nevertheless, the treatment outcomes for refractory epilepsy remain unsatisfactory,^7^ Consequently, it holds significant social and practical importance to investigate the pathogenesis and progression mechanisms associated with epileptic disorders, while striving to identify novel targets for drug development.

It is widely acknowledged that the fundamental mechanism of epilepsy involves alterations in neuronal excitability, which is intricately associated with energy metabolism. Inhibiting pathways related to energy metabolism has been proven effective in ameliorating epileptic discharges and seizures.^8^^,^^9^ Mitochondria exhibit dynamic processes of continuous fission and fusion, collectively known as mitochondrial dynamics.^10^^,^^11^ In a healthy state, mitochondrial dynamics maintain a delicate balance. However, exposure to various stimuli can disrupt this equilibrium, leading to an imbalance in mitochondrial dynamics. Mitochondrial fusion compensates for partially damaged mitochondrial function, while mitochondrial fission ensures overall quality control by generating new mitochondria and eliminating damaged ones.^12^^,^^13^ Therefore, preserving a harmonious mitochondrial dynamic balance is crucial for maintaining optimal mitochondrial function and quality control.^14^^,^^15^

Dysfunction in mitochondrial fission has been linked to the onset and progression of epilepsy.^16^, ^17^, ^18^ Dynamin-related protein 1 (DRP1) is a crucial regulator involved in mitochondrial division.^19^^,^^20^ Studies have revealed that abnormal DRP1 can impair mitochondrial fission, leading to disruptions in ATP synthesis and abnormal energy metabolism.^21^^,^^22^ In animal models of seizures, increased mitochondrial division has been observed, and inhibiting excessive division effectively alleviates seizures and mitigates neuronal damage.^23^^,^^24^ Our previous study has demonstrated that following seizures, mitochondrial fusion protein (MFN1 and MFN2) expression is up-regulated in rats.^25^ Additionally, it was found that PGC-1α can modulate the expression of neuroprotective mitochondria fusion proteins. Inhibition of the AMPK/PGC-1α signaling pathways may increase susceptibility to epileptic seizures in rats. Furthermore, abnormal DRP1 can lead to dysfunctional mitochondrial division.^26^ Inhibiting DRP1 function not only ameliorates excessive mitochondrial division but also reduces the severity of epileptic seizures in experimental rats. Mdivi-1 is a selective inhibitor primarily targeting DRP1 by suppressing its GTPase activity to prevent unwarranted mitochondrial division.^27^^,^^28^ Previous studies have indicated that the use of Mdivi-1 significantly mitigates mitochondrial damage in animal models of epileptic seizures.^29^, ^30^, ^31^

Under normal physiological conditions, dysfunctional and damaged mitochondria produced by mitochondrial fission are eliminated via regular autophagy to maintain mitochondrial homeostasis.^32^ However, when mitochondrial dynamics become imbalanced, leading to excessive division, it can result in the abnormal accumulation of numerous damaged mitochondria. If these are not promptly and effectively removed, they can cause significant harm to the body.^33^^,^^34^ Nevertheless, in cases of excessive mitochondrial division, the body initiates mitophagy which selectively removes abnormally aggregated nonfunctional or damaged mitochondria for lysosomal degradation via an autophagic mechanism.^35^^,^^36^ However, mitophagy has a dual role and can be pathogenic, if either overactivated or inhibited.^37^ In its natural state, mitophagy has a neuroprotective effect on nerve cells.^38^ Nevertheless, studies on animal models of epileptic seizures have shown that mitophagy becomes overactivated during seizures.^39^^,^^40^ Conversely, examination of hippocampal tissue samples from patients with refractory epilepsy has revealed the abnormal accumulation of numerous damaged mitochondria around hippocampal neurons accompanied by neuronal death. This phenomenon may be associated with excessive mitochondrial division and weakened mitophagy during seizures.^41^ Recent studies have shown that excessive inhibition or deficiency of mitochondrial autophagy function can lead to epilepsy.^42^^,^^43^

Vacuolar protein sorting 13D (VPS13D) belongs to the VPS13 family, which consists of four members in humans: VPS13A, B, C, and D.^44^ Studies have revealed that VPS13A and VPS13C function as lipid transporters at the endoplasmic reticulum–mitochondrial membrane contacts, whereas VPS13B is involved in maintaining Golgi integrity and neural axonal growth.^45^ Consequently, the human VPS13 proteins hold significant biomedical importance. VPS13D, located on chromosome 1p36, is a ubiquitin-binding protein with 70 exons spanning a genome size of 280 kb. It consists of 4388 amino acids and has a substantial molecular weight of 492 KD; hence, it is classified as a giant protein.^46^ Studies have shown that VPS13D possesses a ubiquitin-associated domain essential for mitochondrial clearance in *Drosophila*.^47^ In *Drosophila*, VPS13D interacts with DRP1 to regulate mitochondrial autophagy and clearance, with mutations in VPS13D leading to abnormal mitochondrial clearance and autophagy. In humans, mutations in *Vps13d* have been linked to autosomal recessive ataxia, resulting in severe morphological and functional defects in mitochondria.^48^ Additionally, gene sequencing has identified associations between VPS13D mutations and various neurological disorders, such as spastic paraplegia, chorea, and dystonia.^49^ Recent studies have revealed that VPS13D localizes to the membrane contacts of various organelles under different metabolic conditions. These include endoplasmic reticulum–mitochondria/peroxisome membrane contacts under normal conditions and mitochondria–lipid droplet membrane contacts during starvation.^50^ Moreover, VPS13D plays a vital role in the negative regulation of endoplasmic reticulum–mitochondrial membrane contacts. Inhibition of VPS13D leads to extensive tethering between the endoplasmic reticulum and mitochondria, which can be considerably mitigated by inhibiting tethering proteins. Furthermore, VPS13D inhibition significantly alters mitochondrial morphology and distribution, along with severe defects in mitochondrial DNA synthesis.^51^^,^^52^ VPS13D plays a critical role in regulating mitochondrial dynamics and mitophagy.

An increasing understanding of VPS13D's involvement in mitochondrial dynamics and autophagy regulatory mechanisms has led to more studies on this protein. Nevertheless, its specific role in experimental rat epileptic seizures, particularly in regulating mitochondrial dynamics or autophagy, is yet to be explored. Building on our previous study, we propose the scientific hypothesis that VPS13D plays a crucial role in the occurrence of epileptic seizures. We posit that under certain epileptogenic factors, the aberrant functioning of VPS13D disrupts neuronal excitability by affecting mitochondrial dynamics and autophagy, ultimately leading to the development of epileptic seizures. In this study, we aimed to investigate the influence of VPS13D on behavior in a rat model of acute epileptic seizures.

## Material and methods

### Animals

Animal experiments were approve by approve by the Ethics Committee of Zunyi Medical University, and performed using healthy male Sprague Dawley (SD) rats (approximately 8 weeks old) obtained from Changsha Tianqin Biotechnology Co., Ltd. (License: SCXK (Xiang) 2018-0014). The rats were given access to food and water freely and were housed in specific pathogen-free conditions, including a 12-h light/dark cycle, 55.5% humidity, and a temperature of 25 °C. Treatment of animals was strictly as per the “Guidance on Treating Experimental Animals” by Zunyi Medical University. After a one-week adaptation period, the rats were randomly divided into six groups as follows: the Con group (intraperitoneal injection, 0.9% saline, *n* = 5), the EP group (lithium chloride-pilocarpine was used to induce epileptic seizures, and tissues were harvested at 24 h; *n* = 5), the *Vps13d*-kd-LV group (the *Vps13d* gene knockdown lentivirus was injected into the hippocampus of rats via stereotactic technique; subsequently, epileptic seizures were induced by lithium chloride-pilocarpine; *n* = 5), the LV vector group (the empty lentivirus vector was injected into hippocampus of rats via stereotactic technique; subsequently, epileptic seizures were induced by lithium chloride-pilocarpine; *n* = 5), the Mdivi-1 group (Mdivi-1 at a dose of 1.2 mg/kg was intraperitoneally administered, followed by the induction of acute epileptic seizures by lithium chloride-pilocarpine; *n* = 5), and the DMSO group (a solution containing 0.05% DMSO was administered intraperitoneally, followed by the induction of acute epileptic seizures via lithium chloride-pilocarpine; *n* = 5).

### Establishment of a rat model of acute epileptic seizures^53^

Lithium chloride (127 mg/kg) was intraperitoneally administered, followed by intraperitoneal administration of pilocarpine (50 mg/kg) approximately after 18–20 h. Atropine was administered 30 min before pilocarpine injection. The latency period of the initial seizure and frequency of seizures per unit time (1 h) were recorded. Subsequently, diazepam (10 mg/kg) was intraperitoneally administered to terminate seizures. According to the Racine rating standard,^54^ the success of modeling was assessed, with those achieving a grade 4–5 considered as successful models, while those failing to meet this criterion were deemed unsuccessful in modeling. Consequently, all rats that did not meet the modeling standard were excluded from the experimental group. Our previous study^25^^,^^26^ demonstrated that the expression of mitochondrial dynamin-related proteins peaked 24 h after seizure onset. Thus, we selected all tissues collected at this time point after the successful establishment of acute epileptic seizures.

### Stereotactic brain surgeries

The shRNA sequence targeting the *Vps13d* gene was designed using CRISPR/CAS9 technology by the Hefei, China, Xinghuo Liaoyuan Company. The specific sequence for the *Vps13d* SH-2 was 5′-GGGAGAACCATATTGGTAT-3′. The lentivirus (2 μL) was bilaterally injected into the hippocampus of anesthetized rats fixed to a rat stereotaxic apparatus at coordinates AP (3.72 mm), ML (2.0–2.2 mm), and DV (2.8–3.0 mm). Following the injection, the trocar was left in place for 5–10 min to prevent reflux, and the microinjection needle was carefully removed without observing any bleeding for several minutes. Finally, the drilled hole was sealed with dental cement, the surgical area was sterilized again, and layer-by-layer suturing of the incision was performed.

### Behavioral observations

The degree of seizures was assessed using the Racine rating scale. Initially, the time from the termination of pilocarpine injection to the onset of Racine-grade-4-or-higher seizures was recorded as the latency time (min), and the frequency of seizures per unit time (1 h) was recorded.

### Collection of tissue samples

Rats were anesthetized with 1% pentobarbital (0.1 mL/100 g), and the brains were quickly extracted; the hippocampal tissue was rapidly dissected on ice. After that, the brain tissue was placed in tin foil, flash-frozen in liquid nitrogen, and kept at −80 °C for following use.

### Cultivation of cells and establishment of stable cell lines

Mouse hippocampal neuron cell line HT22 cells (Cat. no. 358041; BeNa Culture Collection, Henan, China) were cultured in Dulbecco's modified Eagle's medium with 10% fetal bovine serum and 1% penicillin–streptomycin solution (Invitrogen, Thermo Fisher Scientific, Waltham, USA). The cells were maintained in a 5% CO_2_ incubator at 37 °C (Thermo Fisher Science, Waltham, USA). HT22 cells were infected with the lentivirus suspension, and successfully infected cells were identified and selected using puromycin screening and fluorescence microscopy. The cells were randomly allocated into five groups: the Con group (no treatment; *n* = 5), LV-vector group (transfection of empty lentivirus vector into cells, followed by screening; *n* = 5), *Vps13d*-kd-LV group (knockdown of *Vps13d* gene and transfection of lentivirus into cells, followed by screening; *n* = 5), LV-vector + 3-MA group (transfection of empty lentivirus vector into cells, followed by screening, treated with 5 mmol/L 3-methyladenine for 24 h; *n* = 5), and the *Vps13d*-kd-LV + 3-MA group (knockdown of *Vps13d* gene and transfection of lentivirus into cells, followed by screening, treated with 5 mmol/L 3-methyladenine for 24 h; *n* = 5).

### Quantitative reverse transcription PCR

Primer sequences were synthesized by GENERAL BIOSYSTEMS (Anhui, China), and total RNA was extracted from the cells in each group using the TRIzol method (Sigma–Aldrich, Germany). A NanoDrop 2000 (Thermo Fisher Scientific, Waltham, MA, USA) was used to measure the concentration and purity of RNA. Using real-time PCR equipment (BIORAD) and the HiScript III RT SuperMix for quantitative PCR (+gDNA wiper) (Vazyme R323-01), total RNA was reverse-transcribed into cDNA. The analysis was conducted using AceQ Universal SYBR qPCR Master Mix (Vazyme Q511-02), and the results were determined using the 2^−△△CT^ method.

### Immunohistochemical staining

Sections (approximately 5 microns in thickness) were dewaxed using dimethyl benzene and dehydrated with an ethanol gradient. Subsequently, a double-water hydration process was executed. The slides were washed three times with phosphate buffer saline solution (PBS) and then incubated with 3% H_2_O_2_ for 15 min. Then, a goat serum-blocking solution was applied at 37 °C for 30 min. Primary antibodies VPS13D (1:500, bs-12766R, Bioss antibodies, Beijing, China) and LC3B (1:300, ab62721, Anti-LC3A/B antibody, Abcam, Shanghai, China) were then added to the slides. The slides were stored in a refrigerator at 4 °C for overnight incubation. The cells were then warmed at 37 °C for 30 min and further incubated at room temperature for 30 min using pika universal secondary antibody. Cells were visualized using diaminobenzidine, counterstained with hematoxylin, and fixed for observation.

### Immunofluorescent staining

After perfusion, the rats were euthanized, and their brains were preserved for 24–48 h in 4% paraformaldehyde solution. The brain samples were next dehydrated for 24 h in a 20% sucrose solution, and then for a further 24 h in a 30% sucrose solution, until all of the brain tissue was submerged. The frozen sections were prepared using a cryostat (Lecia/CM1950, USA) and mounted onto glass slides. Alternatively, HT22 cells were seeded on cover slides, gently rinsed with PBS, and fixed with a 4% paraformaldehyde solution for 15 min. After three additional washes with PBS, all slides were incubated in goat serum for 1 h before overnight incubation with the corresponding primary antibody at room temperature. The primary antibody was an anti-VPS13D antibody (bs-12766R, Bioss antibodies, Beijing, China). After washing with PBS, the slides were incubated with a diluted fluorescent secondary antibody (A23420, Abbkine Scientific Co., Ltd, Wuhan, China) at room temperature in the dark for 1 h. HT22 cells were stained with DAPI in darkness for 10 min and then fixed using an anti-fluorescence quenching solution before visualization under a fluorescence microscope.

### Western blot analysis

Using various treatments, total protein was extracted from the cells or tissues using RIPA lysis buffer (P0013, Beyotime Biotechnology, Shanghai, China), which contained PMSF (ST506, Beyotime Biotechnology) and a protease inhibitor. Following centrifugation, the supernatants were collected, and a BCA kit (PC0020, Solarbio, Beijing, China) was used to determine the protein contents. Using 8%, 10%, or 12% SDS-PAGE, equal volumes of denatured proteins were separated and then deposited onto polyvinylidene difluoride membranes (ISEQ00010, Merck KGaA, Darmstadt, Germany). The membranes were incubated at 4 °C in TBST buffer containing 0.05% Tween-20 and 5% BSA or skim milk overnight, as per the manufacturer's instructions. The samples were incubated with secondary antibodies at room temperature for 1 h following three TBST washes the following day. Immunoblotting was performed using the ECL kit (WBKLS0100; Millipore, Billerica, Massachusetts USA), and primary antibodies against LC3B, Drp1, and p62 were used.

### Quantitative proteomics analysis

The knockdown of HT22 in *Vps13d* cells was assessed using proteomic analysis with the PTM-BIO protocol (PTM-Biolabs Co. Ltd, Hangzhou, China). HT22 cells from the normal control and VPS13D knockdown groups were lysed on ice with western and immunoprecipitation lysate (P0013, Beyotime) for 30 min. To obtain the supernatant, the lysates were centrifuged for 30 min at 12,000 *g* and 4 °C. A portion of the supernatant was mixed with magnetic beads and resuspended, followed by protein A/G beads. After washing with PBS, VPS13D-resistant shaking was performed at 4 °C for 2 h. Magnetic suction was used to remove PBS, followed by reoccupation with PBS. After adding the leftover supernatant, the mixture was shaken and left below 4 °C for the entire night. Subsequently, the protein solution was reduced with 5 mM dithiothreitol at 56 °C for 30 min and alkylated with 11 mM iodoacetamide at room temperature for 15 min in darkness. By adding 100 mM TEAB, the protein samples were diluted to a urea concentration of less than 2 M. Afterwards, trypsin was added to the protein for the first overnight digestion at a data mass ratio of 1:50 and for the second 4-h digestion at a data mass ratio of 1:100. Finally, the peptides were desalted on a C18 solid-phase extraction column. The Agilent 300 Extend C18 column (5-micron particles, 4.6 mm ID, and 250 mm in length; Agilent Technologies Co., Ltd., Santa Clara, CA, USA) was used for high pH reversed-phase high-performance liquid chromatography (HPLC) to grade trypsin peptides. Peptide mass spectrometry was performed using Q Exactive Plus Hybrid Quadrupole Orbitrap (Thermo Fisher Scientific). Subsequently, liquid chromatography–mass spectrometry (LC/MS/MS) was performed online with the HPLC system Q Exactive Plus (Thermo Fisher Scientific). The MaxQuant Search engine (v.1.6.15.15.0) was used to process the acquired MS/MS data. Tandem mass spectra were compared to the reverse bait database's 20422 entries in the human SWISS-PROT database.

### Bioinformatics analysis

The biological processes, molecular roles, and cellular components of proteins that exhibited differential expression were examined using the Gene Ontology (GO) database. Utilizing the Kyoto Encyclopedia of Genes and Genomes (KEGG) database, pathway enrichment analysis was carried out. A comparison was made between the enrichment of all detected proteins and the proteins that were differentially expressed.

### Statistical analysis

Mean and standard deviation calculations were used for all continuous variables, the *t*-test and/or analysis of variance were employed to evaluate the differences between groups, statistical analyses were performed using SPSS 19.0 (Version 19.0; IBM Corp., Armonk, NY, USA), and alpha = 0.05 was chosen as the significant criterion. The figures were produced using GraphPad Prism 8 software (GraphPad Company, San Diego, CA, USA).

## Results

### VPS13D expression in the hippocampal tissue of the model SD rats was lower than that of normal SD rats

Immunohistochemical analysis revealed the expression of VPS13D in hippocampal neurons within the CA1, CA3, and dentate gyrus regions, which was predominantly localized in the cytoplasmic compartment of nerve cells (Fig. 1A). Immunofluorescence staining further confirmed the presence of VPS13D in the rat hippocampus's CA1 and CA3 regions and cortical neurons (Fig. 1C). Notably, a significant reduction in VPS13D expression was observed in the epileptic seizure model group compared with that in the control group (*P* < 0.05) (Fig. 1B, D).

**Figure 1:**
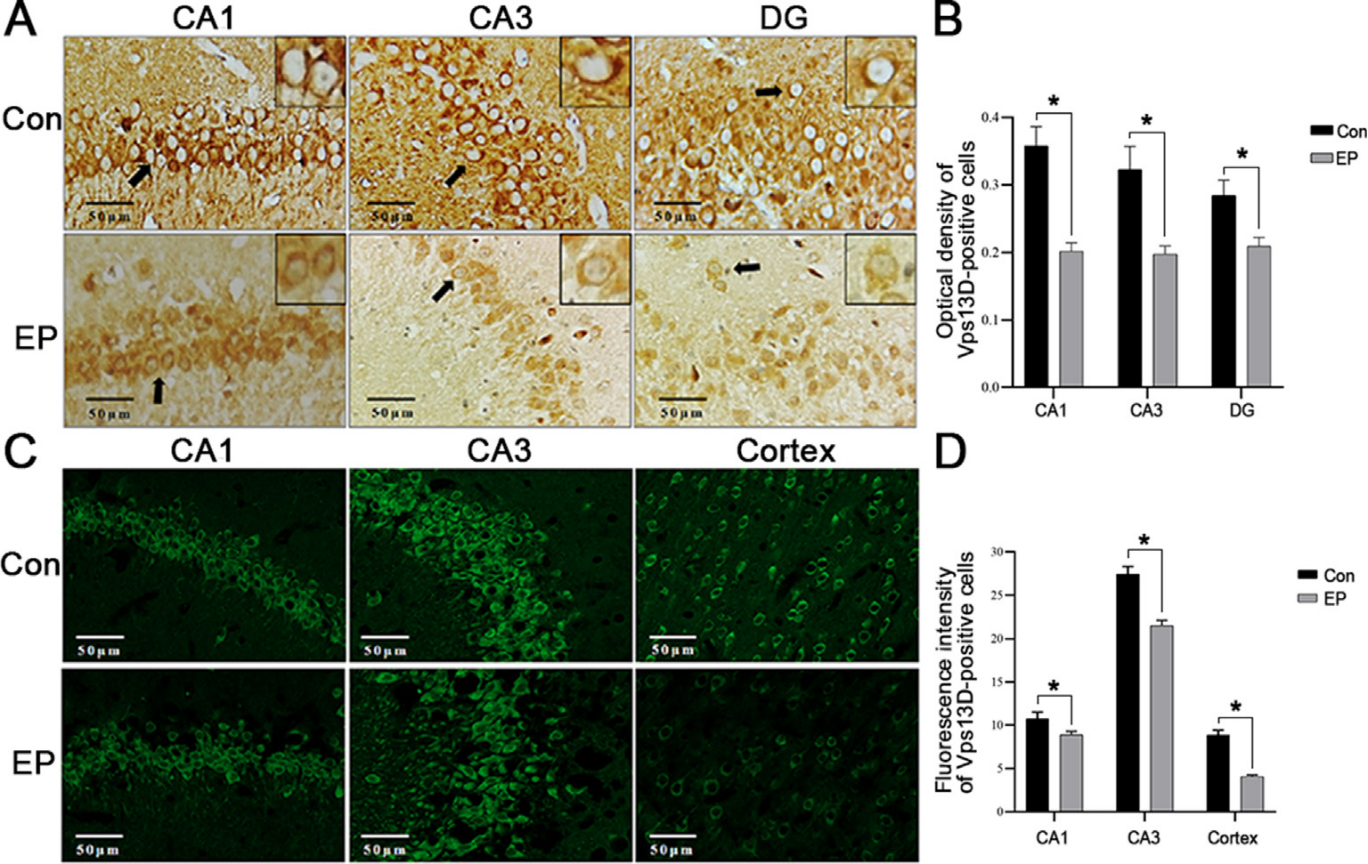
Expression of VPS13D in normal and epileptic Sprague Dawley rats. **(A, B)** Immunohistochemical observation of the optical density of VPS13D-positive cells in the CA1, CA3, and dentate gyrus (DG) regions of Con and EP. Three independent fields were evaluated and reproduced in three Con and three EP rats, with one field of view per segment and up to five segments per organoid were imaged blinded, and the images were analyzed using ImageJ. ∗*P* < 0.05 was considered significant. The data represent the mean±standard deviation and were analyzed by the variance. **(C, D)** Immunofluorescence was used to observe the fluorescence intensity of VPS13D-positive cells in CA1, CA3, and cortical areas of Con and EP. With one field of view per section and up to five sections per organoid were imaged blinded, the fluorescence intensity positive cells was determined per image field, images were analyzed using ImageJ. ^∗^*P* < 0.05 was considered significant. The data represent the mean±standard deviation and were analyzed by the variance.

### Lentivirus successfully reduced VPS13D expression in SD rats and HT22 cells

Following transfection of the lentivirus into HT22 cells and screening for stably transfected strains, the knockdown efficiency of the four groups of shRNA was assessed using quantitative reverse transcription PCR (Fig. 2A), with SH-2 exhibiting the highest efficiency and was used in all subsequent experiments (Fig. 2B). The success of VPS13D knockdown in intervening HT22 cells was confirmed by semi-quantitative immunofluorescence (Fig. 2C, D). The *Vps13d* knockdown lentivirus was stereotaxically injected into the hippocampus of SD rats, and successful virus transfection was confirmed by immunohistochemistry (Fig. 2E, F).

**Figure 2:**
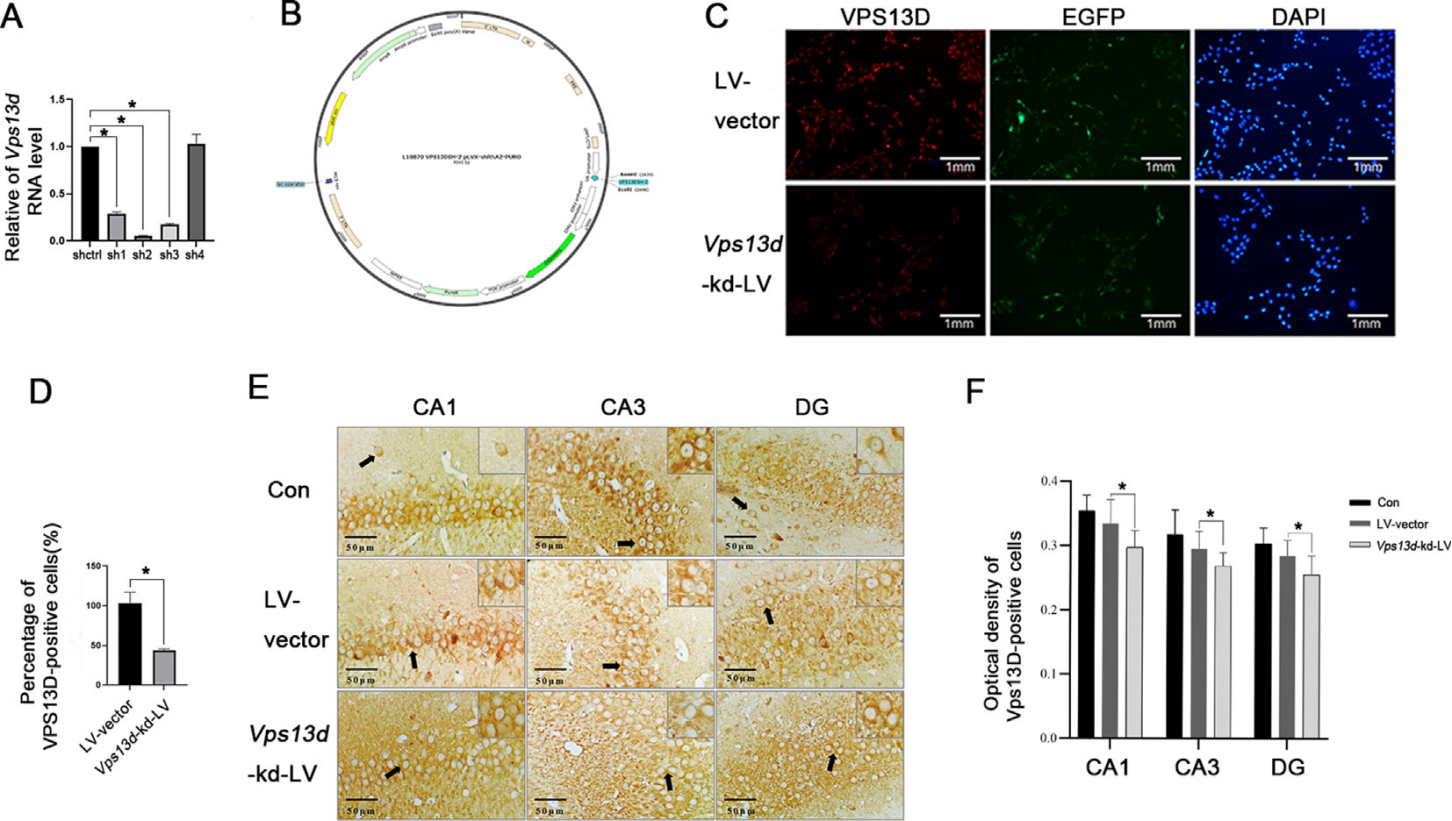
Verification of the intervention efficiency of VPS13D. Quantitative reverse transcription PCR detection of knockdown efficiency of four groups of shRNA: SH-1: GGGTTCAATACTATTT, sH-2: GGGAACACATATTGGTAT, sH-3: GGATCTCACAAGAGAGAAT, and SH-4: GGGCTCCATTCAGATTGAA (**A, B**). Immunofluorescence was used to observe the fluorescence intensity of VPS13D-positive cells of LV-vector and *Vps13d*-kd-LV. Three batches of cells were grown, each consisting of 5 slides, the percentage of VPS13D-positive cells was determined for each image field, and the images were analyzed using ImageJ. After viral intervention, the VPS13D fluorescence intensity of HT22 decreased significantly (**C, D**). ∗*P <0.05* was considered significant. The data represent the mean±standard deviation, and were analyzed by t-test. Immunohistochemical observation of the optical density of VPS13D-positive cells in the CA1, CA3, and dentate gyrus (DG) regions of Con, LV-vector and *Vps13d*-kd-LV. Three independent fields were assessed and reproduced in three Con, LV-vector and *Vps13d*-kd-LV rats, with one field of view per section and up to five sections per organoid were imaged blinded, images were analyzed using ImageJ (**E, F**). ∗*P <0.05* was considered significant. The data represent the mean±standard deviation and were analyzed by the variance.

### Behavioral observations demonstrated that VPS13D exerted a protective effect in the model rats

The latency period of epileptic seizures was significantly reduced in the *Vps13d* knockdown rats, thereby leading to a noticeable increase in seizure frequency per unit time. Additionally, treatment with Mdivi-1 significantly delayed the incubation period of epileptic seizures and significantly reduced seizure frequency per unit time. These differences were statistically significant when compared with the epileptic seizure model, control, LV-vector, and DMSO groups (Fig. 3).

**Figure 3:**
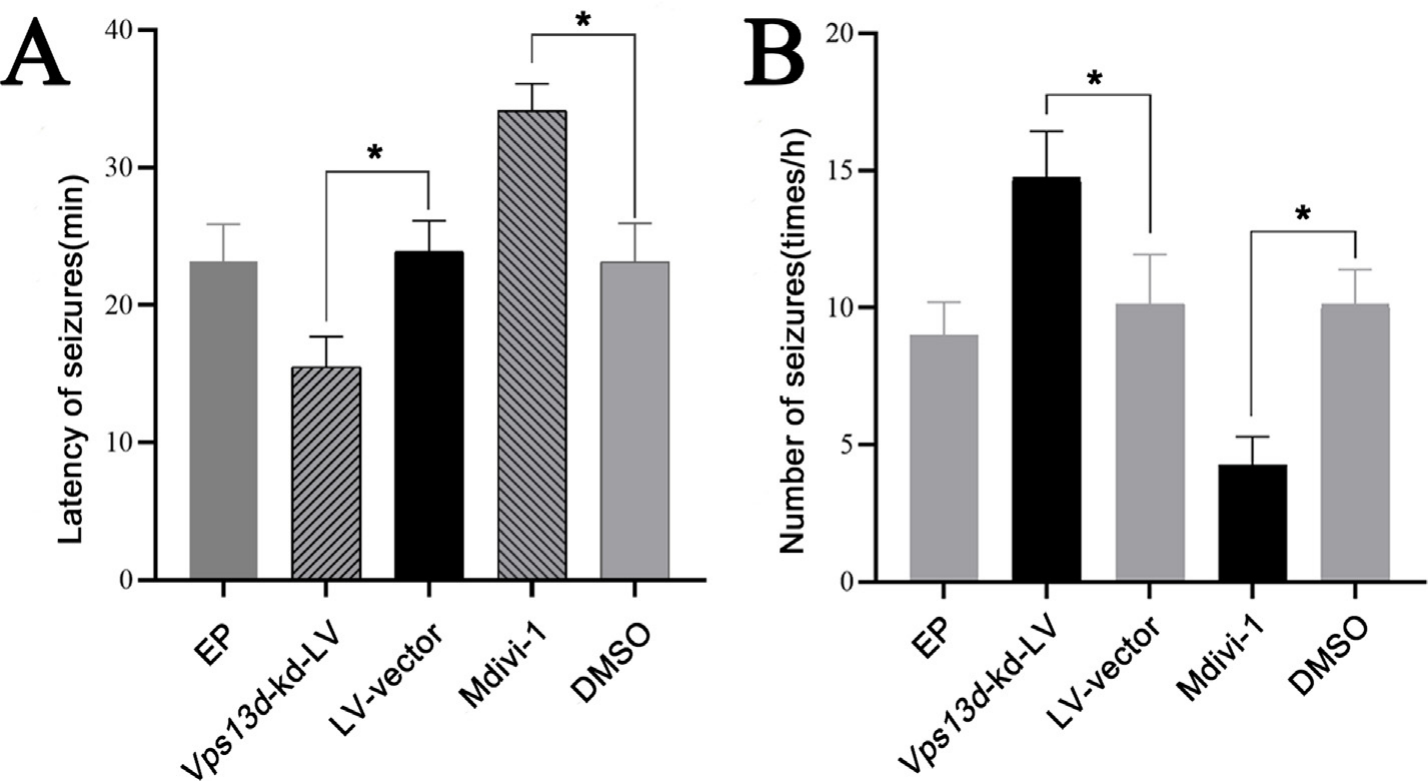
Effect of intervention with VPS13D on delay and frequency of first seizure in each group of rats. **(A)** Epileptic rats displayed a shortened latency of seizures after *Vps13d* knockdown. ^∗^*P* < 0.05 was considered significant, *n* = 5. The data represent the mean±standard deviation, and were analyzed by analysis of variance. **(B)** Epileptic rats displayed an increased frequency of seizures after *Vps13d* knockdown. ^∗^*P* < 0.05 was considered significant, *n* = 5. The data represent the mean±standard deviation, and were analyzed by analysis of variance.

### The proteomic analysis demonstrated significant up-regulation of P62 expression in HT22 cells following *Vps13d* knockdown

The distribution of the identified peptide length was verified to comply with the quality control criteria, demonstrating consistent repeatability of samples and exhibiting satisfactory distribution and variation in protein intensity values among samples (Fig. 4). Liquid chromatography–tandem mass spectrometry was used to perform a quantitative proteomic analysis on the isolated proteins. A fold change >1.2 or <0.833 and *P* <0.05 were used as the criteria for screening differentially expressed proteins between the normal control and *Vps13d* knockdown groups, identifying 86 differentially expressed proteins. Among these, the expression levels of 44 proteins were up-regulated, and the expression levels of 42 proteins were down-regulated (Table 1). Notably, Sqstm1 (P62) expression increased in the *Vps13d* knockdown group (Fig. 5).

**Figure 4:**
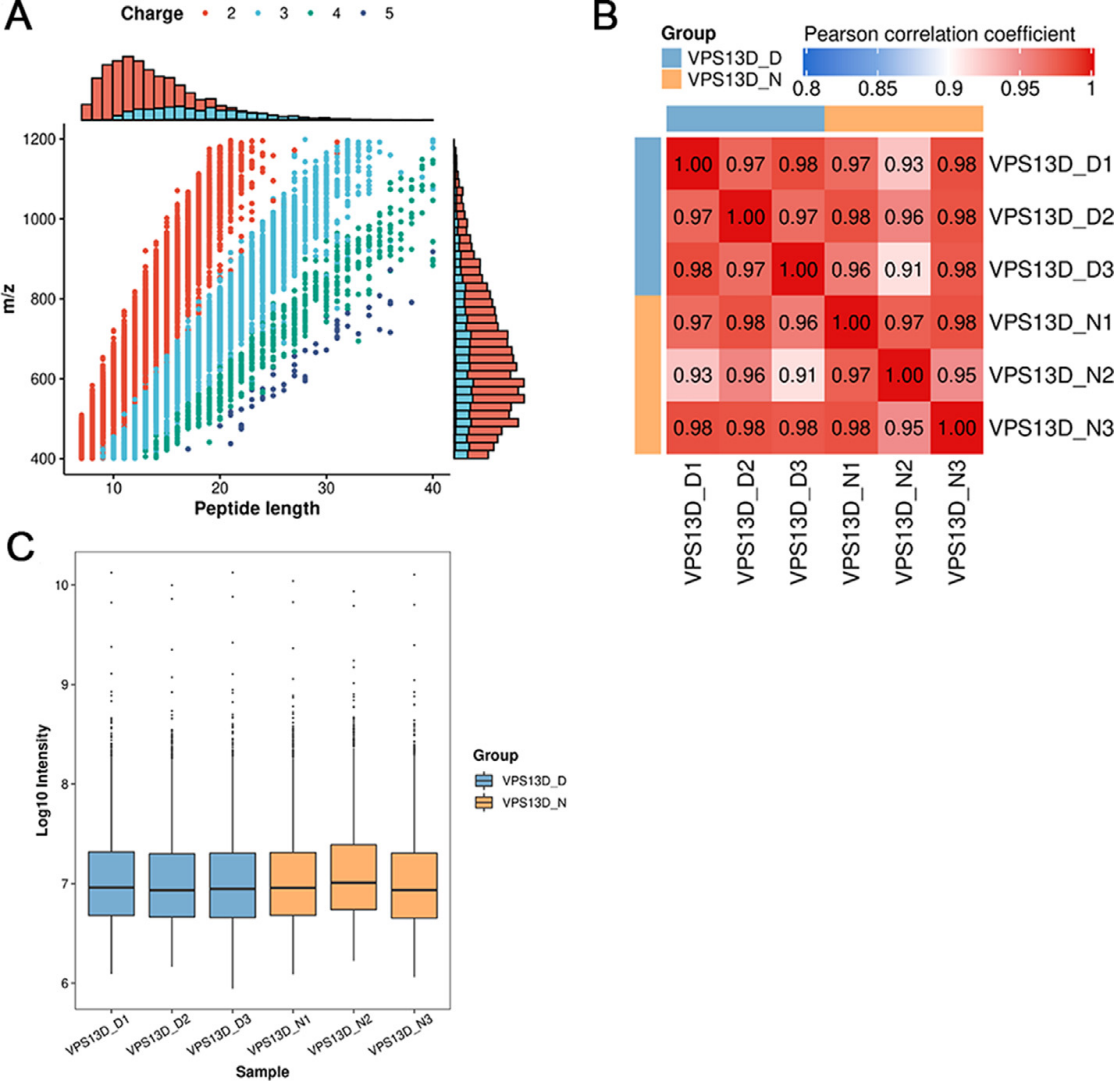
High reliability and repeatability of quantitative proteomics research data. **(A)** Most peptide segments are distributed between 7–20 amino acids, according to a general pattern based on enzymatic hydrolysis and mass spectrometry fragmentation. **(B)** The heat map was plotted using Pearson correlation coefficients between all samples in pairs. **(C)** The box plot of protein intensity values for all samples. *n* = 3. The data were analyzed by Pearson's correlation coefficient. Note：VPS13D_D (VPS13D knockdown group), VPS13D_N (control group).

**Table 1:** List of the differentially expressed proteins.

Group	Protein accession (UniProt accession)					
Up-regulated	O08553	O35345	O70251	P08003	P09528	P10404
	P11440	P16332	P17918	P23116	P24452	P27612
	P31938	P32233	P38647	P43883	P47226	P48678
	P52293	P59325	P62196	P70362	Q03145	Q5XJY5
	Q61599	Q61838	Q62351	Q64133	Q64337	Q6P1B1
	Q6P2B1	Q80X90	Q8BJ71	Q8BMA6	Q91VI7	Q922R8
	Q99KK7	Q99LX0	Q9CQT1	Q9CZ44	Q9D0F9	Q9EQK5
Down-regulated	A2ASS6	C0HKE6	P10922	P11103	P28659	P49312
	P51410	P61255	P61358	P62245	P62274	P62806
	P62889	P67984	P84228	P97351	Q62095	Q6ZWU9
	Q6ZWV3	Q80U78	Q80XU3	Q8BG05	Q8BP71	Q8BSY0
	Q8BVY0	Q8K2F8	Q8R081	Q8VC70	Q8VEE4	Q91YN9
	Q91YT7	Q921F2	Q9CX86	Q9CZX8	Q9D0I8	Q9D0T1
	Q9D7N3	Q9D7S7	Q9D903	Q9EP72	Q9EPU4	Q9JJI8

**Figure 5:**
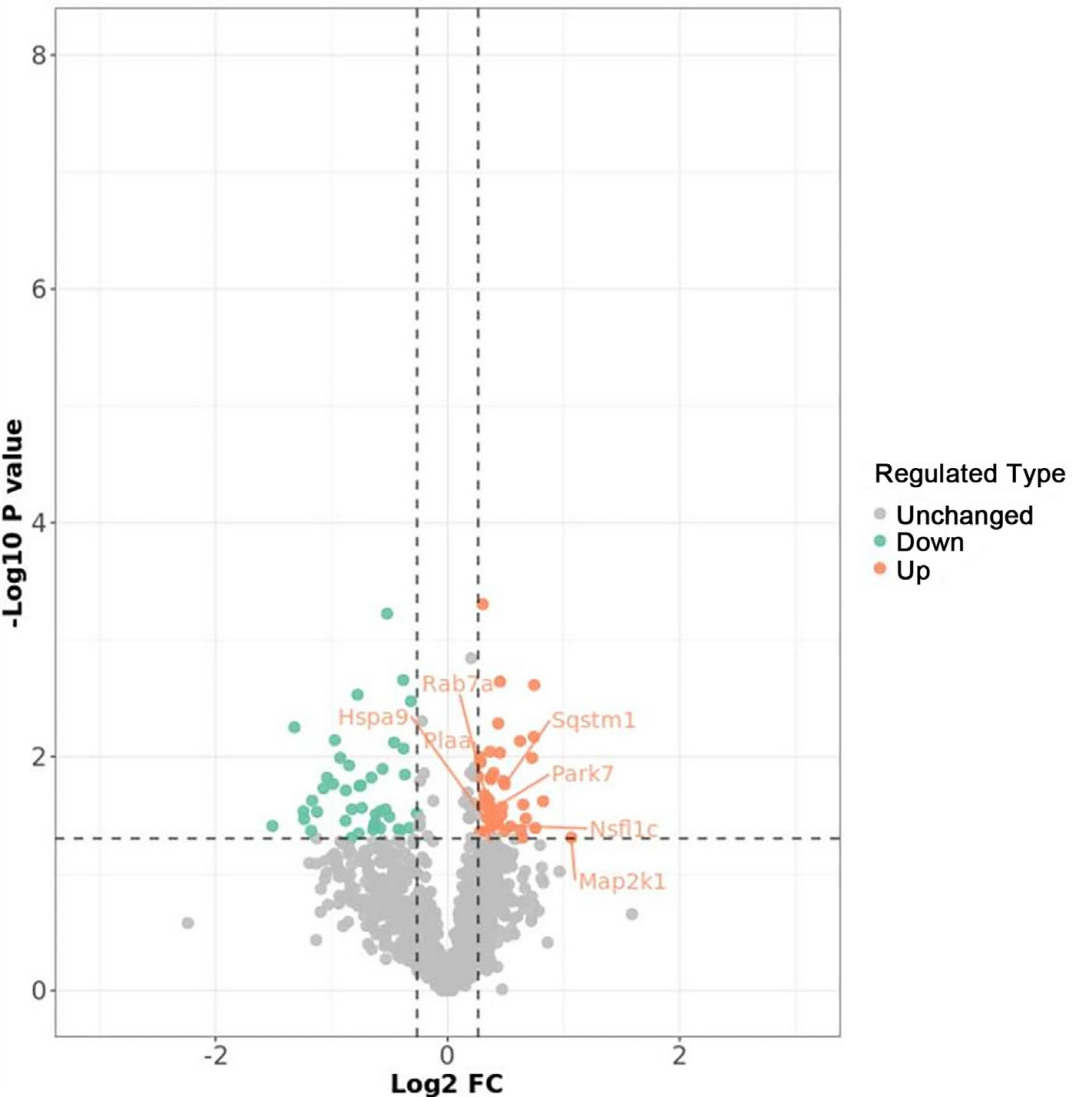
The volcano plot of differential protein analysis. A significant increase in differential protein expression is indicated by red dots, a significant decrease in differential protein expression is indicated by green dots, and gray dots indicate no significant difference.

### *Vps13d* knockdown induces alterations in mitochondrial morphology in HT22 cells

The morphology of the mitochondria in cells from different treatment groups was observed under a transmission electron microscope (scale ruler: 250 nm), with the red arrows indicating the mitochondria. Compared with the control and LV-vector groups, the cells of the *Vps13d* knockdown group exhibited irregular mitochondrial shapes, blurred mitochondrial membrane edges, damaged mitochondrial cristae, and swollen and fragmented mitochondria (Fig. 6).

**Figure 6:**
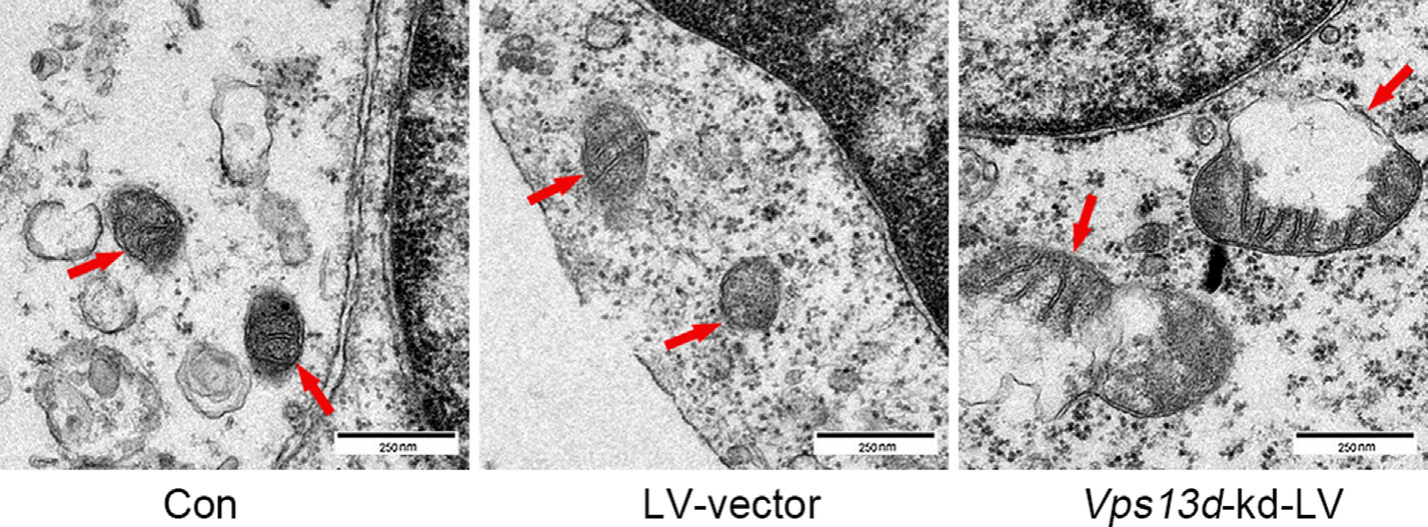
The mitochondrial morphology of HT22 after *Vps13d* knockdown observed by projection electron microscopy. The *Vps13d* knockdown group showed irregular mitochondrial morphology, blurred mitochondrial margins, damaged mitochondrial cristae, and mitochondrial swelling and fragmentation.

### *Vps13d* knockdown increases mitochondrial fission and autophagy inhibition in SD rats

Western blot analysis revealed an increase in DRP1 expression in the epileptic group compared to the control group, and a significant increase in DRP1 expression in the *Vps13d* knockout group compared to the LV-vector groups (*P* < 0.05) (Fig. 7A, B), indicating an up-regulation of mitochondrial fission. Subsequently, to confirm the involvement of mitochondrial division in autophagy and assess the impact of selective inhibitors, such as DRP1 Mdivi-1, we observed that the ratio of LC3II/LC3I significantly decreased in the *Vps13d* knockdown group and Mdivi-1 compared with that in the LV-vector and DMSO groups (Fig. 7C, D). Furthermore, compared with the control, LV-vector, and DMSO treatment groups, there was a notable increase in P62 expression levels in the *Vps13d* knockdown group and the Mdivi-1 groups (Fig. 7E, F). These findings suggest that reducing VPS13D levels increases mitochondrial fission and attenuates autophagy in SD rats.

**Figure 7:**
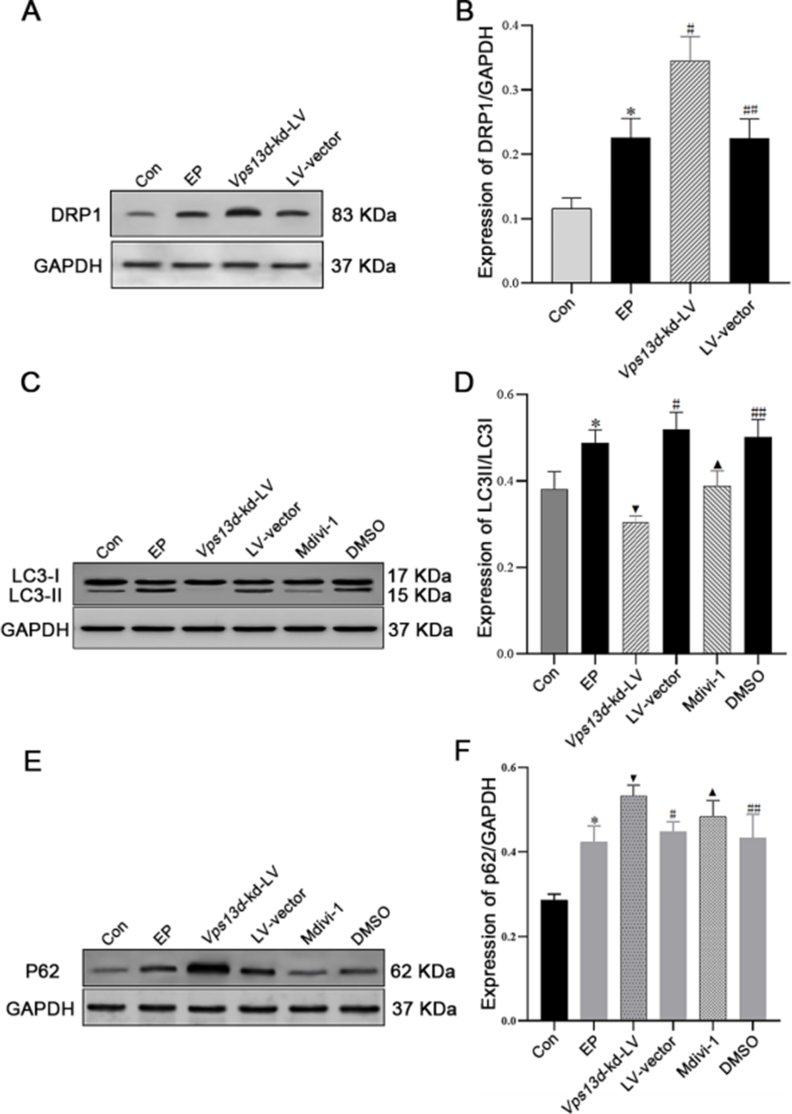
Knockdown of VPS13D promotes mitochondrial division and inhibits autophagy in rats. **(A)** After knockdown intervention in *Vps13d*, western blot assays were used to detect mitochondrial division-related protein DRP1 expression. **(B)** The expression of DRP1 significantly increased after intervention in *Vps13d*. ∗*P* < 0.05 versus the Con group; ^#^*P* < 0.05 versus the LV-vector group; ^##^*P* > 0.05 versus the EP group; *n* = 5; data were analyzed by analysis of variance. **(C)** After using the DRP1 inhibitor Mdivi-1, the expression of the autophagy-related protein LC3II was detected using western blot assays. **(D)** The expression of LC3II/LC3I was significantly decreased after intervention in *Vps13d*. ∗*P* < 0.05 versus the EP group; ^▼^*P* < 0.05 versus the LV-vector group; ^▲^*P* < 0.05 versus the DMSO group; ^#^*P* > 0.05 versus the EP and DMSO group; *n* = 5; data were analyzed by analysis of variance. **(E)** After using the DRP1 inhibitor Mdivi-1, the expression of the autophagy-related protein P62 was detected using western blot assays. **(F)** The expression of P62 was significantly increased after intervention in *Vps13d*. ∗*P* < 0.05 versus the EP group; ^▼^*P* < 0.05 versus the LV-vector group; ^▲^*P* < 0.05 versus the DMSO group; ^#^*P* > 0.05 versus the EP and DMSO group; *n* = 5; data were analyzed by analysis of variance.

### *Vps13d* knockdown in HT22 cells leads to increased mitochondrial fission and inhibited autophagy

Immunofluorescence analysis revealed that, compared with the LV-vector group, the *Vps13d* knockdown group had decreased expression of LC3 in the cell sets (*P* < 0.05) (Fig. 8A, B). Western blot analysis further demonstrated that compared with the normal control and LV-vector groups, knockdown of *Vps13d*, treatment with 3-MA, or combined knockdown of *Vps13d* and 3-MA led to increased expression levels of P62 and DRP1 (*P* < 0.05) (Fig. 8C–F). These findings suggest that VPS13D induction in HT22 cells leads to an increase in mitochondrial fission and a decrease in autophagy.

**Figure 8:**
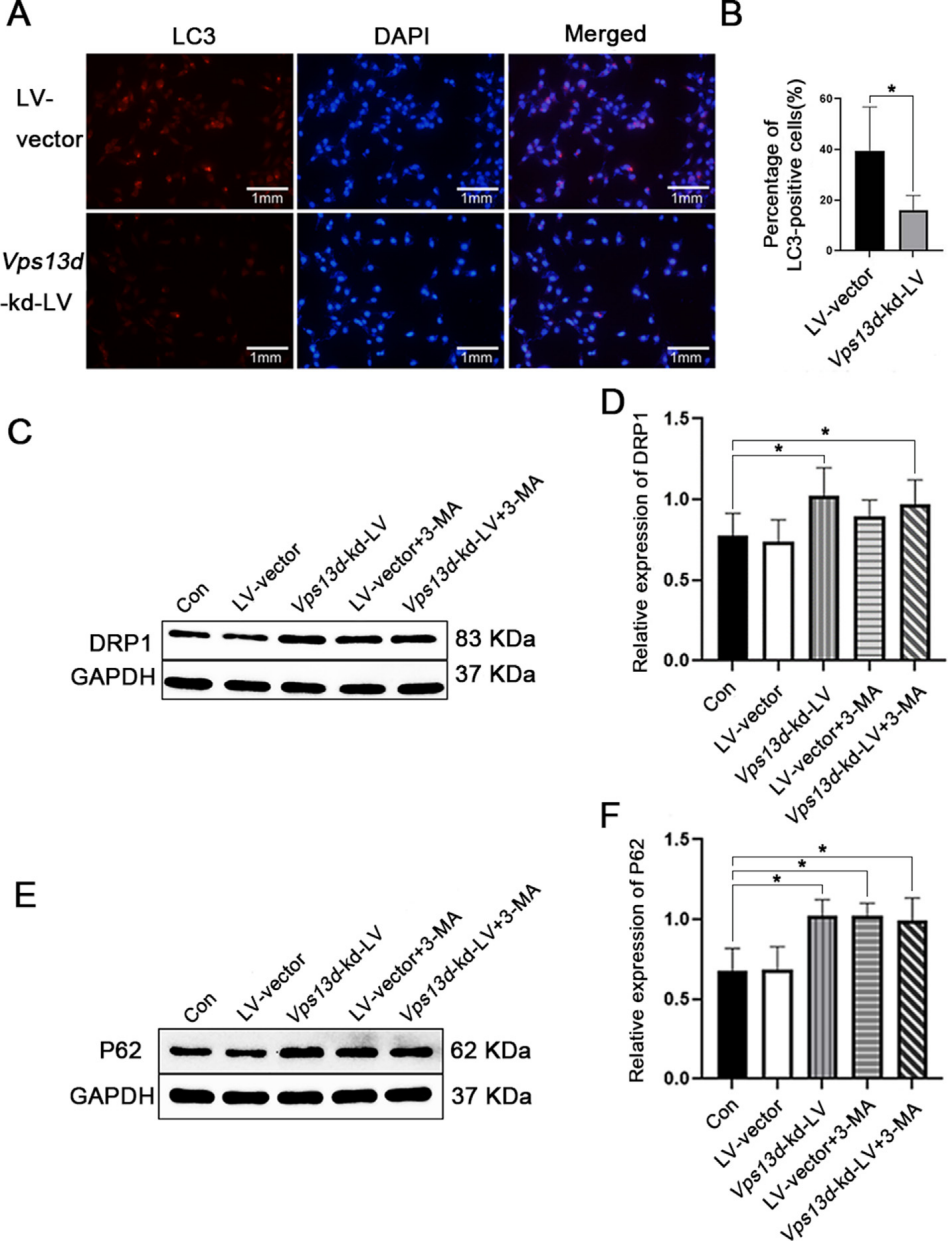
The knockdown of *Vps13d* in HT22 cells promotes mitochondrial fission and inhibits autophagy. **(A)** After chronic viral intervention, the expression of LC3 in HT22 was observed using immunofluorescence microscopy. **(B)** Compared with the LV-vector group, the *Vps13d* knockdown group had decreased expression of LC3 in the cell sets. ^∗^*P* < 0.05; *n* = 5; data were analyzed by *t*-test. **(C)** Western blot detection of DRP1 expression in HT22 after lentivirus intervention and use of autophagy inhibitor 3-MA. **(D)** Western blot analysis demonstrated increased levels of DRP1 after treatment with the autophagy inhibitor 3-MA. ^∗^*P* < 0.05 versus the LV-vector group; ^∗∗^*P* < 0.05 versus the LV-vector group; ^#^*P* > 0.05 versus the *Vps13d*-kd-LV and LV-vector-3MA group; *n* = 5; data were analyzed by analysis of variance. **(E)** Western blot detection of P62 expression in HT22 after lentivirus intervention and use of autophagy inhibitor 3-MA. **(F)** Western blot analysis demonstrated increased levels of P62 after treatment with the autophagy inhibitor 3-MA. ^∗^*P* < 0.05 versus the LV-vector group; ^∗∗^*P* < 0.05 versus the LV-vector group; ^#^*P* > 0.05 versus the *Vps13d*-kd-LV and LV-vector-3MA group; *n* = 5; data were analyzed by analysis of variance.

## Discussion

Our findings highlight a distinct difference in VPS13D expression between normal rats and those with acute epileptic seizures. Knockdown of *Vps13d* led to reduced seizure latency and increased seizure frequency in the experimental rats. Mass spectrometry analysis revealed the influence of *Vps13d* on autophagy, whereas our molecular biology analyses indicated that *Vps13d* gene knockdown results in a dual effect, excessive mitochondrial division, and marked inhibition of autophagy. This excessive division leads to the accumulation of damaged mitochondria due to impaired removal, thereby creating a detrimental cycle in which a surplus of dysfunctional mitochondria accumulates, thus significantly increasing neuronal excitability and toxic effects, ultimately leading to seizures.

In our study, we used the widely recognized and extensively used horse Roca acute epileptic seizure model to study seizure mechanisms. VPS13D is a macromolecular substance with a molecular weight of 492 KD, synthesized from polypeptide segments. However, complete verification by western blotting remains challenging.^44^ Therefore, we employed immunohistochemistry and immunofluorescence techniques to localize and semi-quantitatively analyze VPS13D at the cellular level. Immunohistochemistry analysis revealed that VPS13D was predominantly expressed in the cytoplasm of nerve cells in the hippocampus's CA1, CA3, and dentate gyrus regions. The semi-quantitative analysis demonstrated significantly lower optical density values of VPS13D-positive cells in the hippocampus of acute epileptic seizure model rats than those of the control group. The results also indicated that the expression of VPS13D in the CA1, CA3, and cortical regions of the hippocampus predominantly colocalized with neuronal markers. Semi-quantitative analysis of the VPS13D fluorescence signal intensity revealed a significant decrease in absolute values in the epileptic seizure model group compared with those in the control group. Immunohistochemistry and immunofluorescence analyses further confirmed a significant reduction in VPS13D expression in the brain tissue of epileptic seizure model rats. Therefore, we propose that VPS13D plays a role in epileptogenesis and potentially acts as a protective factor. However, the functional significance of this differential expression remains unclear.

Therefore, we employed the CRISPR/CAS9 technology to generate lentiviruses capable of knockdown *Vps13d* expression and lentiviruses carrying empty viral sequences. Subsequently, we employed quantitative reverse transcription PCR to confirm and evaluate the efficacy of VPS13D knockdown. The selected *Vps13d* gene knockdown virus was then stereotaxically injected into rat brain tissue. We performed an immunohistochemistry analysis to assess the impact of the *Vps13d* knockdown virus transfection. The results revealed a significant reduction in the optical density of VPS13D expression in the hippocampus of the gene knockdown group. Furthermore, we performed behavioral assessment in experimental rats and revealed a significant reduction in the incubation period of epileptic seizures in *Vps13d* gene knockdown rats accompanied by a notable increase in seizure frequency per unit of time. These findings strongly suggest that VPS13D played a role in epileptic seizures in our experimental rat model. However, the specific underlying mechanism remains unknown.

We performed quantitative proteomic analysis on HT22 cells following *Vps13d* knockdown and compared them with normal HT22 cells. Mass spectrometry revealed that VPS13D primarily regulates DNA synthesis, gene expression, and protein translation in mice (Figs. S1–S3), with seven autophagy-related proteins exhibiting significant differences. Notably, the autophagy substrate protein P62 expression exhibited a substantial increase, indicating suppression of autophagy after *Vps13d* knockdown. Furthermore, RAB7A protein expression was elevated in the *Vps13d* knockdown group, suggesting its crucial role in mediating autophagosome–lysosome fusion and implying that the mechanism by which VPS13D inhibits autophagy may be located upstream of this fusion process.

In our previous study, we observed increased expression of mitochondrial fusion proteins after seizures in rats. PGC-1α was identified to play a neuroprotective role by regulating the expression of mitochondrial fusion proteins and suppressing the AMPK/PGC-1α signaling pathway. We have also demonstrated that the abnormal mitochondrial DRP1 can result in mitochondrial division dysfunction. As such, inhibiting DRP1 function reduces excessive mitochondrial division and mitigates the severity of epileptic seizures in rats. However, a previous study has indicated that VPS13D interacts with DRP1 to regulate mitochondrial clearance and autophagy in *Drosophila* and that mutations in *Vps13d* may lead to abnormal mitochondrial autophagy.^47^ Therefore, we hypothesized that VPS13D may also be involved in seizure activity by affecting both mitochondrial fission and autophagy.

To determine the impact of *Vps13d* knockdown on mitochondrial dynamics, western blot analysis was used to assess the expression of mitochondrial DRP1 in the hippocampus of *Vps13d* knockdown rats. Our findings revealed a significant increase in the DRP1 protein level in the hippocampus of *Vps13d* gene knockdown rats, with a relatively pronounced upward trend compared with that in non-intervention epileptic seizure model rats. Additionally, we used HT22 cells to establish a *Vps13d* knockdown HT22 cell strain via viral intervention. To assess the impact of this intervention, we examined DRP1 expression following *Vps13d* knockdown using western blot analysis. The results indicated a significant increase in the DRP1 protein levels associated with mitochondrial dynamics. Our findings demonstrate that the knockdown of *Vps13d* affects the expression of the mitochondrial DRP1 both *in vitro* and *in vivo*. Based on previous studies, we propose that *Vps13d* knockdown promotes excessive mitochondrial fission. However, prior studies have indicated that excessive mitochondrial fission can lead to the abnormal accumulation of numerous nonfunctional or damaged mitochondria.^26^^,^^37^ Under such circumstances, only normal mitophagy can efficiently eliminate these dysfunctional mitochondria promptly; otherwise, they may cause significant harm to the organism.

Therefore, we investigated whether *Vps13d* knockdown affected autophagy following gene intervention. To assess the impact of *Vps13d* knockdown on autophagy, we used western blot analysis to examine the expression levels of LC3II, an autophagy marker protein, and P62, an autophagy adaptor protein, in the brain tissues of experimental rats following *Vps13d* knockdown. LC3 is widely recognized as a crucial indicator of autophagy activity, with LC3II levels and the LC3II/LC3I ratio serving as reliable indicators. Reduced LC3II expression and a decreased LC3II/LC3I ratio signify weakened autophagic activity, while increased expression suggests an enhanced autophagic response. P62 plays a pivotal role in mediating autophagic degradation, and its expression increases when autophagic function declines. Our study results demonstrated that *Vps13d* knockdown significantly reduced the expression of LC3II, resulting in a reduced LC3II/LC31 ratio, while the expression of the autophagy adaptor protein P62 significantly increased. In addition, we cultured mouse hippocampal neuron cell line HT22 *in vitro* and treated it with a lentivirus targeting *Vps13d* to establish a stable *Vps13d* knockdown HT22 cell line. Immunofluorescence was employed to assess the expression of LC3 following the *Vps13d* knockdown. Western blot analysis revealed a significant reduction in VPS13D levels after the intervention accompanied by an increase in P62 expression and a notable decrease in the proportion of LC3-positive cells in the *Vps13d* knockdown group. Our findings from both *in vitro* and *in vivo* experiments collectively demonstrate that the intervention-induced reduction of the *Vps13d* gene impacts the expression of the autophagy marker protein LC3II and its interaction with the P62 protein. Consequently, we propose that *Vps13d* knockdown leads to excessive inhibition of autophagy. Furthermore, in HT22 cells, we observed an up-regulation of DRP1 expression following both *Vps13d* knockdown and intervention with the autophagy inhibitor 3-MA. However, there was no significant increase in DRP1 expression after the combined intervention of *Vps13d* knockdown and 3-MA. Our study results demonstrated that VPS13D may modulate mitochondrial dynamics by suppressing the autophagy pathway.

Our study has some limitations. First, our *in vitro* experiments did not reveal any differences in the expression of mitochondrial fusion-related proteins, thus hindering our understanding of the changes in mitochondrial dynamics related to fusion. Second, we did not measure the mitochondrial membrane potential. Hence, we could not confirm the relationship between alterations in mitochondrial potential and their effects on neuronal excitability, as well as neuronal apoptosis and necrosis. Third, we did not explore the interaction between mitophagy and mitochondrial dynamics following VPS13D intervention. Additionally, we did not investigate the specific mechanisms involved. As a result, we cannot provide a detailed explanation of the upstream relationships between these processes. Finally, we did not perform further experimental studies to investigate the relationship between autophagosomes and lysosomes after *Vps13d* knockdown. Consequently, we lack a comprehensive understanding of the mechanism underlying mitochondrial autophagy following VPS13D intervention.

## Conclusions

Our findings highlight the neuroprotective effects of VPS13D in epileptic seizure model rats, as knocking down the *Vps13d* gene increased seizure frequency and reduced seizure latency in these rats. Furthermore, molecular biology and mass spectrometry analyses proved that *Vps13d* gene knockdown results in a dual phenomenon: excessive mitochondrial division and marked inhibition of autophagy function. Consequently, we propose that the *Vps13d* gene plays a crucial role in regulating seizures in rats, and its mechanism may involve modulation of mitochondrial division and autophagy function by influencing mitochondrial dynamics.

## Author contributions

Jian Wang and Fan Zhang conceived the study and designed the experiments. Jian Wang and Fan Zhang performed the experiments, analyzed the data, and produced the figures. Jian Wang and Fan Zhang drafted the manuscript and they contributed equally to this work. Zhong Luo and Haiqing Zhang provided critical input and expertise and revised the manuscript. Changyin Yu, and Zucai Xu revised and approved the final manuscript.

## Conflict of interests

The authors have no conflict of interests to declare.

## Funding

This work was supported by the Science and Technology Fund Project of the Guizhou Provincial Health Commission (China) (No. gzwkj2023-109, gzwkj2021.017, gzwjkj2020-1-010), the Science and Technology Plan Project of Zunyi City, Guizhou, China (No. ZSKHZC-HZ(2020)172), the Science and Technology Project in Guizhou Province, China (No. QKHJC-ZK[2021] NO.408), and the 10.13039/501100001809National Natural Science Foundation of China (No. 82101527).

## Data availability

The original contributions presented in this study are included in the article, and further inquiries can be directed to the corresponding authors.

## Figures and Tables

**Figure S1:**
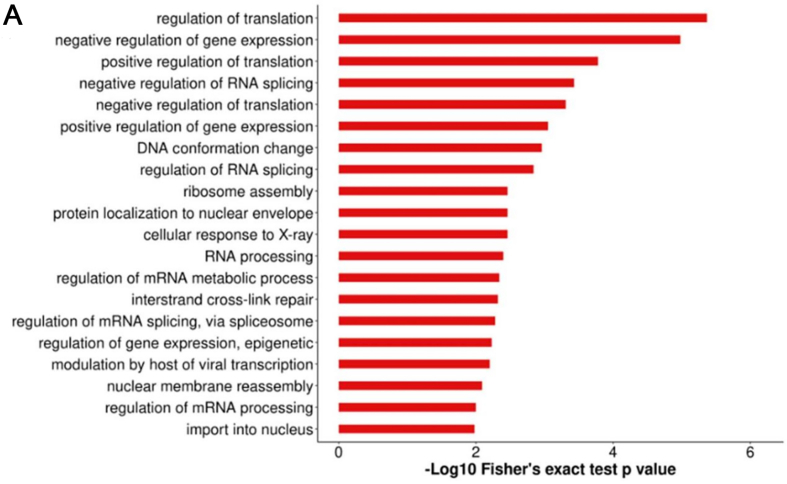
Bar charts for the GO enrichment analysis of differentially expressed proteins. **(A)** The top biological processes of the differentially expressed proteins included regulation of translation, negative regulation of gene expression, regulation of RNA splicing, positive gene expression regulation, DNA conformational changes, and RNA processing. **(B)** In terms of molecular function, the main enriched proteins were mRNA 3′-UTR binding proteins, nucleic acid binding proteins, DNA binding proteins, and RNA binding proteins. **(C)** Regarding cell composition, the main enriched proteins were nucleic acid–protein complexes, cytoplasmic ribosomes, non-membrane-bound organelles, ribosome subunits, and ribosomes.

**Figure S1B:**
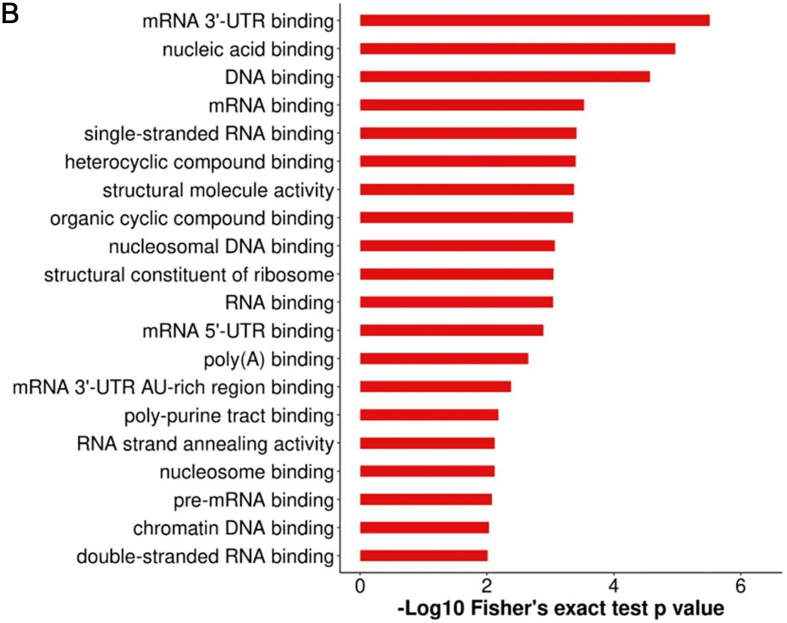


**Figure S1C:**
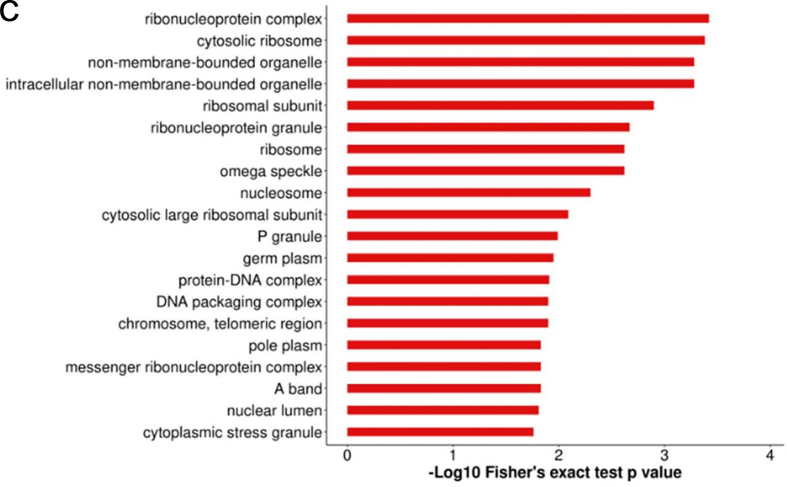


**Figure S2:**
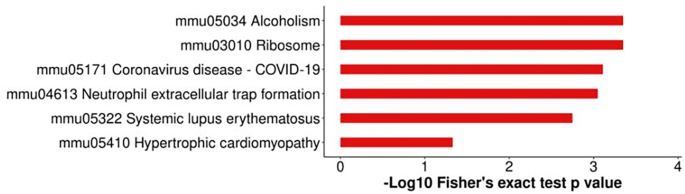
The bar graph presenting the KEGG pathway enrichment analysis results for the differentially expressed proteins. Alcoholism, ribosome, novel coronavirus pneumonia, neutrophil extracellular trap formation, systemic lupus erythematosus, and hypertrophic cardiomyopathy are significantly enriched pathways.

**Figure S3:**
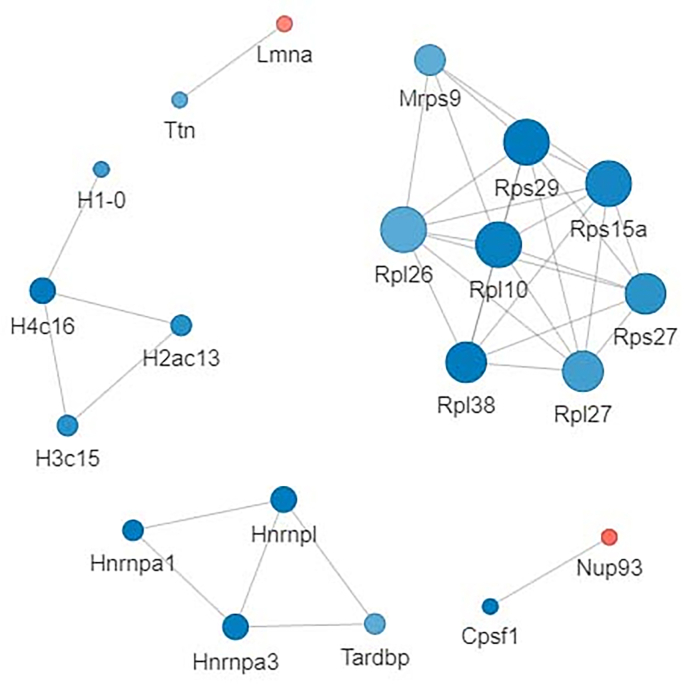
Direct interaction network of the differentially expressed proteins. Circles indicate differential proteins, blue indicates proteins with reduced expression in *Vps13d* knockdown cells, and red indicates proteins with increased expression in *Vps13d* knockdown cells. The darker the color, the greater the degree of difference. The size of the dots indicates the number of proteins that interact with them. Proteins with significant interactions, such as Rpl10, Rps29, Rps15a, Rpl38, and H4c16, may be key proteins in VPS13D.
